# Some Properties of Weighted Tsallis and Kaniadakis Divergences

**DOI:** 10.3390/e24111616

**Published:** 2022-11-05

**Authors:** Răzvan-Cornel Sfetcu, Sorina-Cezarina Sfetcu, Vasile Preda

**Affiliations:** 1 Faculty of Mathematics and Computer Science, University of Bucharest, Str. Academiei 14, 010014 Bucharest, Romania; 2 “Gheorghe Mihoc-Caius Iacob” Institute of Mathematical Statistics and Applied Mathematics, Calea 13 Septembrie 13, 050711 Bucharest, Romania; 3 “Costin C. Kiriţescu” National Institute of Economic Research, Calea 13 Septembrie 13, 050711 Bucharest, Romania

**Keywords:** Tsallis logarithm, Kaniadakis logarithm, weighted Tsallis divergence, weighted Kaniadakis divergence

## Abstract

We are concerned with the weighted Tsallis and Kaniadakis divergences between two measures. More precisely, we find inequalities between these divergences and Tsallis and Kaniadakis logarithms, prove that they are limited by similar bounds with those that limit Kullback–Leibler divergence and show that are pseudo-additive.

## 1. Introduction

Shannon entropy, in the form we know it, was introduced by Boltzmann and used by Shannon in the context of Information Theory. This entropy has applications in Statistical Thermodynamics, Combinatorics and Machine Learning. In Machine Learning, Shannon entropy represents the basis for building decision trees and fitting classification models.

In the last couple of years, many generalizations of Shannon entropy appeared: Tsallis entropy, Kaniadakis entropy, Rényi entropy, Varma entropy, weighted entropy, relative entropy, cumulative entropy, etc. These entropies have applications in areas such as Physics, Information Theory, Probabilities, Communication Theory, and Statistics.

Tsallis entropy was introduced by C. Tsallis in [1] and is applied to: Income distribution (see [2,3]), Internet (see [4]), Non-coding human DNA (see [5]), Plasma (see [6]), and Stock exchanges (see [7,8]).

Kaniadakis entropy was introduced by G. Kaniadakis in [9] and is useful in many areas like: Finance (see [10,11,12]), Astrophysics (see [13,14]), Networks (see [15,16]), Economics (see [17,18]), and Statistical Mechanics (see [19,20,21]).

S. Kullback and R.A. Leibler were concerned “to measure” “the distance” or “the divergence” between statistical populations and they generalized Shannon entropy by defining, in [22], a nonsymmetric measure, called Kullback–Leibler divergence. This divergence between two probability measures $\mu_{1}$ and $\mu_{2}$ on a measurable non-negligible set *A* is additive, non-negative and greater than $\log\left(\frac{\mu_{1}\left(A\right)}{\mu_{2}\left(A\right)}\right)$, where log is the classical logarithm function. Divergences are a key tool in Information Geometry (see [23]).

The goodness of fit test is based on the Corrected Weighted Kullback–Leibler divergence (see [24]) and, as a consequence, it inherits all special characteristics of this divergence measure. Narowcki and Harding proposed the use of weighted entropy as a measure of investment risk (see [25]). Afterwards, Guiaşu used the weighted entropy to group data with respect to the importance of specific regions of the domain (see [26]), Di Crescenzo and Longobardi propose the weighted residual and past entropies (see [27]) and Suhov and Zohren proposed the quantum version of weighted entropy and its properties in Quantum Statistical Mechanics (see [28]).

Working with the Kullback–Leibler divergence formula and using the same technique like in the cases of Tsallis and Kaniadakis entropies (i.e., classical logarithm is replaced by Tsallis logarithm, respectively, by Kaniadakis logarithm), Tsallis and Kaniadakis divergences were introduced in some papers (see [29,30,31,32]).

Motivated by the aforementioned facts and by the papers [33,34,35], we deal with the weighted Tsallis and Kaniadakis divergences in this article.

In the following, we briefly describe the structure of the paper. Section 2 is dedicated to preliminaries. In Section 3, using some inequalities concerning the Tsallis logarithm, we obtain inequalities between the weighted Tsallis and Kaniadakis divergences on a non-negligible measurable arbitrary set and Tsallis logarithm, respectively, Kaniadakis logarithm (see Theorem 1). Finally, we prove that the weighted Tsallis and Kaniadakis divergences are limited by bounds that are similar to those that limit Kullback–Leibler divergence (see Theorem 2). In Section 4, we define the weighted Tsallis and Kaniadakis divergences for product measure spaces and prove some pseudo-additivity properties for them (see Theorem 3).

Other interesting results related to the present topics can be found in: [36,37,38,39,40].

## 2. Preliminary Facts

**Definition** **1.** Let $k\in R^{*}$. We consider the Tsallis logarithm given by

$$\log^{T}x=\left\{\begin{array}{cc} \frac{x^{k}-1}{k} & \;\mathrm{if}\;x>0\\ \;\;\;\;0 & \;\mathrm{if}\;x=0 \end{array}\right.$$

*and the Kaniadakis logarithm given via*

$$\log^{K}x=\left\{\begin{array}{cc} \frac{x^{k}-x^{-k}}{2k} & \;\mathrm{if}\;x>0\\ \;\;\;\;\;0 & \;\mathrm{if}\;x=0. \end{array}\right.$$

**Remark** **1.** *It is easy to see that $\log_{k}^{K}x=\frac{1}{2}\left(\log_{k}^{T}x+\log_{-k}^{T}x\right)$ for any x≥0.*

*We have $\lim_{k\to 0}\log_{k}^{T}x=\lim_{k\to 0}\log_{k}^{K}x=\log x$ for any x>0 (“log” is the classical logarithm function).*

**Definition** **2.** *Let (Ω,T) be a measurable space and $\mu ,\nu :T\to {\bar{R}}_{+}=\left[0,\infty \right)\cup \left\{\infty \right\}$ two measures. We say that μ is absolutely continuous with respect to ν if, for any A∈T such that ν(A)=0, one has μ(A)=0.*

**Notation** **1.** *If μ and ν are absolutely continuous with respect to each other, we denote this fact by μ∼ν.*

In the absence of other mentions, we work in the following scenario: Let $\left(\Omega ,T,\mu_{i}\right)$, i=1,2 be two measure spaces and λ a measure on (Ω,T) such that $\mu_{i}∼\lambda$ for any i=1,2. With the help of Radon–Nikodým Theorem we find two non-negative measurable functions $f_{1}$ and $f_{2}$ defined on Ω such that $\mu_{i}\left(A\right)=\int_{A}f_{i}d\lambda$ for any A∈T and any i=1,2. Consider w:Ω→(0,∞) a weight function (i.e., *w* is a non-negative measurable function).

**Definition** **3.** *Let A∈T. The weighted Tsallis divergence on A between $\mu_{1}$ and $\mu_{2}$ is defined via*

$$D_{k}^{w,T}\left(\mu_{1}|\mu_{2},A\right)=\left\{\begin{array}{cc} \frac{1}{\int_{A}wd\mu_{1}}\int_{A}w\log_{k}^{T}\left(\frac{f_{1}}{f_{2}}\right)d\mu_{1} & \;\mathrm{if}\;\mu_{1}\left(A\right)\neq 0\\ \;\;\;\;\;\;\;\;\;\;\;\;\;\;\;0 & \;\mathrm{if}\;\mu_{1}\left(A\right)=0 \end{array}\right.$$

*and the weighted Kaniadakis divergence on A between*$\mu_{1}$ and $\mu_{2}$ *is given by*

$$D_{k}^{w,K}\left(\mu_{1}|\mu_{2},A\right)=\left\{\begin{array}{cc} \frac{1}{\int_{A}wd\mu_{1}}\int_{A}w\log_{k}^{K}\left(\frac{f_{1}}{f_{2}}\right)d\mu_{1} & \;\mathrm{if}\;\mu_{1}\left(A\right)\neq 0\\ \;\;\;\;\;\;\;\;\;\;\;\;\;\;\;0 & \;\mathrm{if}\;\mu_{1}\left(A\right)=0. \end{array}\right.$$

**Remark** **2.** *We assume that all divergences and integrals which appear in this paper are finite.*

**Remark** **3.** *We can see that the values of $D_{k}^{w,T}\left(\mu_{1}|\mu_{2},A\right)$ and $D_{k}^{w,K}\left(\mu_{1}|\mu_{2},A\right)$ do not depend on the choice of reference measure λ (because $\frac{f_{1}}{f_{2}}=\frac{d\mu_{1}/d\lambda }{d\mu_{2}/d\lambda }=\frac{d\mu_{1}}{d\mu_{2}}$).*

## 3. Bounds of the Weighted Tsallis and Kaniadakis Divergences

The proof of the following lemma is elementary and is omitted.

**Lemma** **1.** *For any x∈(0,∞)\{1}, let $\varphi_{x}:R\backslash \left\{0\right\}\to R$,*

$$\varphi_{x}\left(t\right)=\frac{x^{t}-1}{t}.$$

*The function* $\varphi_{x}$ *is strictly increasing.*

The next two corollaries are very useful in this article.

**Corollary** **1.** *Let*x>0 and $k\in \left(-1,\infty \right)\backslash \left\{0\right\}$. *Then,*

$$x\log_{k}^{T}x\geq x-1$$

*and the equality is valid if and only if*x=1.

**Corollary** **2.** *Let x>0 and $k\in \left(-\frac{1}{2},1\right)\backslash \left\{0\right\}$. Then,*

$$\frac{2(\sqrt{x}-1)}{\sqrt{x}}\leq \log_{k}^{T}x\leq x-1.$$

*We have equality in these inequalities if and only if x=1.*

**Theorem** **1.** *Let A∈T such that $\mu_{i}\left(A\right)\neq 0$ for any i=1,2.*

*(a) Assume that $k\in \left(-1,\infty \right)\backslash \left\{0\right\}$. Then,*

$$D_{k}^{w,T}\left(\mu_{1}|\mu_{2},A\right)\geq \log_{k}^{T}\left(\frac{\int_{A}wd\mu_{1}}{\int_{A}wd\mu_{2}}\right).$$

*(b) Assume that k∈(−1,1)\{0}. Then,*

$$D_{k}^{w,K}\left(\mu_{1}|\mu_{2},A\right)\geq \log_{k}^{K}\left(\frac{\int_{A}wd\mu_{1}}{\int_{A}wd\mu_{2}}\right).$$

*In both cases, the equality holds if and only if $\frac{f_{1}\left(\omega \right)}{f_{2}\left(\omega \right)}=\frac{\int_{A}wd\mu_{1}}{\int_{A}wd\mu_{2}}$λ−a.e. for ω∈A.*

**Proof** **.** (a) We will make the proof in two steps.

*Step 1.* Assume that $\int_{A}wd\mu_{1}=\int_{A}wd\mu_{2}$. Because $\log_{k}^{T}\left(\frac{\int_{A}wd\mu_{1}}{\int_{A}wd\mu_{2}}\right)=0$, we have to show that $D_{k}^{w,T}\left(\mu_{1}|\mu_{2},A\right)\geq 0$.

According to Corollary 1, we have

$$D_{k}^{w,T}\left(\mu_{1}|\mu_{2},A\right)=\frac{1}{\int_{A}wd\mu_{1}}\int_{A}w\log_{k}^{T}\left(\frac{f_{1}}{f_{2}}\right)d\mu_{1}=\frac{1}{\int_{A}wd\mu_{1}}\int_{A}w\cdot \frac{f_{2}}{f_{1}}\cdot \frac{f_{1}}{f_{2}}\log_{k}^{T}\left(\frac{f_{1}}{f_{2}}\right)d\mu_{1}\geq \;\frac{1}{\int_{A}wd\mu_{1}}\int_{A}w\cdot \frac{f_{2}}{f_{1}}\left(\frac{f_{1}}{f_{2}}-1\right)d\mu_{1}=\frac{1}{\int_{A}wd\mu_{1}}\int_{A}w\left(1-\frac{f_{2}}{f_{1}}\right)d\mu_{1}=\;1-\frac{1}{\int_{A}wd\mu_{1}}\int_{A}w\cdot \frac{f_{2}}{f_{1}}d\mu_{1}=1-\frac{\int_{A}wd\mu_{2}}{\int_{A}wd\mu_{1}}=0.$$

The equality holds if and only if $\frac{f_{1}\left(\omega \right)}{f_{2}\left(\omega \right)}=1$$\mu_{1}-a.e$, i.e., if and only if $\frac{f_{1}\left(\omega \right)}{f_{2}\left(\omega \right)}=1$λ−a.e.

*Step 2.* Let A∈T with $\mu_{i}\left(A\right)\neq 0$ for any i=1,2. We define the measures ${\tilde{\mu }}_{1}$ and ${\tilde{\mu }}_{2}$ via

$${\tilde{\mu }}_{i}\left(B\right)=\frac{\mu_{i}\left(B\right)}{\int_{A}wd\mu_{i}}\mathrm{for}\;\mathrm{any}\;B\in T\;\mathrm{and}\;\mathrm{any}\;i=1,2.$$

We remark that $\int_{A}wd{\tilde{\mu }}_{1}=\int_{A}wd{\tilde{\mu }}_{2}=1$.

Hence the weighted Tsallis divergence between ${\tilde{\mu }}_{1}$ and ${\tilde{\mu }}_{2}$ on *A* is

$$D_{k}^{T}\left({\tilde{\mu }}_{1}|{\tilde{\mu }}_{2},A\right)=\int_{A}w\cdot \frac{f_{1}}{\int_{A}wd\mu_{1}}\cdot \log_{k}^{T}\left(\frac{\frac{f_{1}}{\int_{A}wd\mu_{1}}}{\frac{f_{2}}{\int_{A}wd\mu_{2}}}\right)d\lambda =\;\int_{A}w\cdot \frac{f_{1}}{\int_{A}wd\mu_{1}}\cdot \log_{k}^{T}\left(\frac{f_{1}\int_{A}wd\mu_{2}}{f_{2}\int_{A}wd\mu_{1}}\right)d\lambda .$$

We deduce from *Step 1* that $D_{k}^{T}\left({\tilde{\mu }}_{1}|{\tilde{\mu }}_{2},A\right)\geq 0$.

So,

$$0\leq \int_{A}w\cdot \frac{f_{1}}{\int_{A}wd\mu_{1}}\cdot \log_{k}^{T}\left(\frac{f_{1}\int_{A}wd\mu_{2}}{f_{2}\int_{A}wd\mu_{1}}\right)d\lambda =\int_{A}w\cdot \frac{f_{1}}{\int_{A}wd\mu_{1}}\cdot \frac{{\left(\frac{f_{1}\int_{A}wd\mu_{2}}{f_{2}\int_{A}wd\mu_{1}}\right)}^{k}-1}{k}d\lambda =\;\int_{A}w\cdot \frac{f_{1}}{\int_{A}wd\mu_{1}}\cdot \frac{{\left(\frac{\int_{A}wd\mu_{2}}{\int_{A}wd\mu_{1}}\right)}^{k}\cdot {\left(\frac{f_{1}}{f_{2}}\right)}^{k}-1}{k}d\lambda =\;\int_{A}w\cdot \frac{f_{1}}{\int_{A}wd\mu_{1}}\cdot \frac{{\left(\frac{\int_{A}wd\mu_{2}}{\int_{A}wd\mu_{1}}\right)}^{k}\cdot \left[{\left(\frac{f_{1}}{f_{2}}\right)}^{k}-1+1\right]-1}{k}d\lambda =\;\int_{A}w\cdot \frac{f_{1}}{\int_{A}wd\mu_{1}}\cdot \left[{\left(\frac{\int_{A}wd\mu_{2}}{\int_{A}wd\mu_{1}}\right)}^{k}\cdot \frac{{\left(\frac{f_{1}}{f_{2}}\right)}^{k}-1}{k}+\frac{{\left(\frac{\int_{A}wd\mu_{2}}{\int_{A}wd\mu_{1}}\right)}^{k}-1}{k}\right]d\lambda =\;{\left(\frac{\int_{A}wd\mu_{2}}{\int_{A}wd\mu_{1}}\right)}^{k}D_{k}^{w,T}\left(\mu_{1}|\mu_{2},A\right)+\frac{{\left(\frac{\int_{A}wd\mu_{2}}{\int_{A}wd\mu_{1}}\right)}^{k}-1}{k}.$$

Hence, $D_{k}^{w,T}\left(\mu_{1}|\mu_{2},A\right)\geq {\left(\frac{\int_{A}wd\mu_{1}}{\int_{A}wd\mu_{2}}\right)}^{k}\cdot \frac{1-{\left(\frac{\int_{A}wd\mu_{2}}{\int_{A}wd\mu_{1}}\right)}^{k}}{k}=\log_{k}^{T}\left(\frac{\int_{A}wd\mu_{1}}{\int_{A}wd\mu_{2}}\right)$.

The equality holds if and only if $\frac{f_{1}\left(\omega \right)}{\int_{A}wd\mu_{1}}=\frac{f_{2}\left(\omega \right)}{\int_{A}wd\mu_{2}}$λ−a.e. for ω∈A, i.e., if and only if $\frac{f_{1}\left(\omega \right)}{f_{2}\left(\omega \right)}=\frac{\int_{A}wd\mu_{1}}{\int_{A}wd\mu_{2}}$λ−a.e. for ω∈A.

(b) Because $\log_{k}^{K}x=\frac{1}{2}\left(\log_{k}^{T}x+\log_{-k}^{T}x\right)$, we obtain

$$D_{k}^{w,K}\left(\mu_{1}|\mu_{2}\right)=\frac{1}{2}\left(D_{k}^{w,T}\left(\mu_{1}|\mu_{2}\right)+D_{-k}^{w,T}\left(\mu_{1}|\mu_{2}\right)\right)\geq \;\frac{1}{2}\left(\log_{k}^{T}\left(\frac{\int_{A}wd\mu_{1}}{\int_{A}wd\mu_{2}}\right)+\log_{-k}^{T}\left(\frac{\int_{A}wd\mu_{1}}{\int_{A}wd\mu_{2}}\right)\right)=\log_{k}^{K}\left(\frac{\int_{A}wd\mu_{1}}{\int_{A}wd\mu_{2}}\right)\left(\mathrm{see}\;\left(a\right)\right).$$

We have equality in the preceding inequality if and only if

$$\frac{1}{2}\left(D_{k}^{w,T}\left(\mu_{1}|\mu_{2},A\right)+D_{-k}^{w,T}\left(\mu_{1}|\mu_{2},A\right)\right)=\frac{1}{2}\left(\log_{k}^{T}\left(\frac{\int_{A}wd\mu_{1}}{\int_{A}wd\mu_{2}}\right)+\log_{-k}^{T}\left(\frac{\int_{A}wd\mu_{1}}{\int_{A}wd\mu_{2}}\right)\right),$$

which is equivalent to $D_{k}^{w,T}\left(\mu_{1}|\mu_{2},A\right)=\log_{k}^{T}\left(\frac{\int_{A}wd\mu_{1}}{\int_{A}wd\mu_{2}}\right)$ and $D_{-k}^{w,T}\left(\mu_{1}|\mu_{2},A\right)=\log_{-k}^{T}\left(\frac{\int_{A}wd\mu_{1}}{\int_{A}wd\mu_{2}}\right)$ and these are equivalent to $\frac{f_{1}\left(\omega \right)}{f_{2}\left(\omega \right)}=\frac{\int_{A}wd\mu_{1}}{\int_{A}wd\mu_{2}}$λ−a.e. for ω∈A.

□

**Theorem** **2.** *Let A∈T such that $\mu_{i}\left(A\right)\neq 0$ for any i=1,2 and $\int_{A}wd\mu_{1}=\int_{A}wd\mu_{2}$.*

*(a) Assume that $k\in \left(-\frac{1}{2},1\right)\backslash \left\{0\right\}$. Then,*

$$\frac{1}{\int_{A}wd\mu_{1}}\int_{A}w{\left(\sqrt{\frac{d\mu_{1}}{d\mu_{2}}}-1\right)}^{2}d\mu_{2}\leq D_{k}^{w,T}\left(\mu_{1}|\mu_{2}\right)\leq \frac{1}{\int_{A}wd\mu_{1}}\int_{A}w{\left(\frac{d\mu_{1}}{d\mu_{2}}-1\right)}^{2}d\mu_{2}.$$

*(b) Assume that $k\in \left(-\frac{1}{2},\frac{1}{2}\right)\backslash \left\{0\right\}$. Then,*

$$\frac{1}{\int_{A}wd\mu_{1}}\int_{A}w{\left(\sqrt{\frac{d\mu_{1}}{d\mu_{2}}}-1\right)}^{2}d\mu_{2}\leq D_{k}^{w,K}\left(\mu_{1}|\mu_{2}\right)\leq \frac{1}{\int_{A}wd\mu_{1}}\int_{A}w{\left(\frac{d\mu_{1}}{d\mu_{2}}-1\right)}^{2}d\mu_{2}.$$

**Proof** **.** (a) We have (see Corollary 2)

$$D_{k}^{w,T}\left(\mu_{1}|\mu_{2}\right)=\frac{1}{\int_{A}wd\mu_{1}}\int_{A}w\cdot \frac{f_{1}}{f_{2}}\cdot \log_{k}^{T}\left(\frac{f_{1}}{f_{2}}\right)d\mu_{2}\geq \;\frac{1}{\int_{A}wd\mu_{1}}\int_{A}w\cdot \frac{f_{1}}{f_{2}}\cdot \frac{2\left(\sqrt{\frac{f_{1}}{f_{2}}}-1\right)}{\sqrt{\frac{f_{1}}{f_{2}}}}d\mu_{2}=\frac{1}{\int_{A}wd\mu_{1}}\int_{A}2w\cdot \sqrt{\frac{f_{1}}{f_{2}}}\cdot \left(\sqrt{\frac{f_{1}}{f_{2}}}-1\right)d\mu_{2}=\;\frac{1}{\int_{A}wd\mu_{1}}\int_{A}2w\cdot \sqrt{\frac{d\mu_{1}}{d\mu_{2}}}\cdot \left(\sqrt{\frac{d\mu_{1}}{d\mu_{2}}}-1\right)d\mu_{2}=\frac{1}{\int_{A}wd\mu_{1}}\int_{A}w\cdot \left(2\cdot \frac{d\mu_{1}}{d\mu_{2}}-2\sqrt{\frac{d\mu_{1}}{d\mu_{2}}}\right)d\mu_{2}=\;\frac{1}{\int_{A}wd\mu_{1}}\int_{A}w\cdot \frac{d\mu_{1}}{d\mu_{2}}d\mu_{2}-2\cdot \frac{1}{\int_{A}wd\mu_{1}}\int_{A}w\cdot \sqrt{\frac{d\mu_{1}}{d\mu_{2}}}d\mu_{2}+\frac{1}{\int_{A}wd\mu_{1}}\int_{A}wd\mu_{2}=\;\frac{1}{\int_{A}wd\mu_{1}}\int_{A}w\cdot {\left(\sqrt{\frac{d\mu_{1}}{d\mu_{2}}}-1\right)}^{2}d\mu_{2}.$$

On the other hand (see again Corollary 2),

$$D_{k}^{w,T}\left(\mu_{1}|\mu_{2}\right)=\frac{1}{\int_{A}wd\mu_{1}}\int_{A}w\cdot \frac{f_{1}}{f_{2}}\cdot \log_{k}^{T}\left(\frac{f_{1}}{f_{2}}\right)d\mu_{2}\leq \frac{1}{\int_{A}wd\mu_{1}}\int_{A}w\cdot \frac{f_{1}}{f_{2}}\cdot \left(\frac{f_{1}}{f_{2}}-1\right)d\mu_{2}=\;\frac{1}{\int_{A}wd\mu_{1}}\int_{A}w\cdot \frac{d\mu_{1}}{d\mu_{2}}\cdot \left(\frac{d\mu_{1}}{d\mu_{2}}-1\right)d\mu_{2}=\frac{1}{\int_{A}wd\mu_{1}}\int_{A}w\cdot \left({\left(\frac{d\mu_{1}}{d\mu_{2}}\right)}^{2}-\frac{d\mu_{1}}{d\mu_{2}}\right)d\mu_{2}=\;\frac{1}{\int_{A}wd\mu_{1}}\int_{A}w\cdot {\left(\frac{d\mu_{1}}{d\mu_{2}}\right)}^{2}d\mu_{2}-2\cdot \frac{1}{\int_{A}wd\mu_{1}}\int_{A}w\cdot \frac{d\mu_{1}}{d\mu_{2}}d\mu_{2}+\frac{1}{\int_{A}wd\mu_{1}}\int_{A}wd\mu_{2}=\;\frac{1}{\int_{A}wd\mu_{1}}\int_{A}w{\left(\frac{d\mu_{1}}{d\mu_{2}}-1\right)}^{2}d\mu_{2}.$$

(b) Using (a) we obtain

$$\frac{1}{2}\cdot \frac{1}{\int_{A}wd\mu_{1}}\int_{A}w{\left(\sqrt{\frac{d\mu_{1}}{d\mu_{2}}}-1\right)}^{2}d\mu_{2}+\frac{1}{2}\cdot \frac{1}{\int_{A}wd\mu_{1}}\int_{A}w{\left(\sqrt{\frac{d\mu_{1}}{d\mu_{2}}}-1\right)}^{2}d\mu_{2}\leq \;\frac{1}{2}D_{k}^{w,T}\left(\mu_{1}|\mu_{2}\right)+\frac{1}{2}D_{-k}^{w,T}\left(\mu_{1}|\mu_{2}\right)\leq \frac{1}{2}\cdot \frac{1}{\int_{A}wd\mu_{1}}\int_{A}w{\left(\frac{d\mu_{1}}{d\mu_{2}}-1\right)}^{2}d\mu_{2}+\frac{1}{2}\cdot \frac{1}{\int_{A}wd\mu_{1}}\int_{A}w{\left(\frac{d\mu_{1}}{d\mu_{2}}-1\right)}^{2}d\mu_{2}.$$

Hence,

$$\frac{1}{\int_{A}wd\mu_{1}}\int_{A}w{\left(\sqrt{\frac{d\mu_{1}}{d\mu_{2}}}-1\right)}^{2}d\mu_{2}\leq D_{k}^{w,K}\left(\mu_{1}|\mu_{2}\right)\leq \frac{1}{\int_{A}wd\mu_{1}}\int_{A}w{\left(\frac{d\mu_{1}}{d\mu_{2}}-1\right)}^{2}d\mu_{2}.$$

□

## 4. Pseudo-Additivity of the Weighted Tsallis and Kaniadakis Divergences

Let $\left(\Omega ,T,\mu_{i}\right)$, i=1,2 be two measure spaces and $\lambda_{1}$ a measure on (Ω,T) such that $\mu_{i}∼\lambda_{1}$ for any i=1,2. We consider Radon–Nikodým derivatives $f_{1}^{\left(1\right)}$ and $f_{2}^{\left(1\right)}$ on Ω, i.e., $f_{i}^{\left(1\right)}=\frac{d\mu_{i}}{d\lambda_{1}}$ for any i=1,2. Let also $\left(S,S,\nu_{j}\right)$, j=1,2 be two measure spaces and $\lambda_{2}$ a measure on (S,S) such that $\nu_{j}∼\lambda_{2}$ for any j=1,2. We apply Radon–Nikodým Theorem and find the non-negative measurable functions $f_{1}^{\left(2\right)}$ and $f_{2}^{\left(2\right)}$ defined on *S* such that $f_{j}^{\left(2\right)}=\frac{d\nu_{j}}{d\lambda_{2}}$ for any j=1,2. We take $w_{1}:\Omega \to \left(0,\infty \right)$ and $w_{2}:S\to \left(0,\infty \right)$ two weight functions.

We consider the measure λ on (Ω×S,T×S) induced by $\lambda_{1}$ and $\lambda_{2}$. Because $\mu_{i}\times \nu_{i}$ is absolutely continuous with respect to λ, we apply Radon–Nikodým Theorem and find two non-negative measurable functions $f_{1}$ and $f_{2}$ on Ω×S such that $f_{i}=\frac{d(\mu_{i}\times \nu_{i})}{d\lambda }$ for any i=1,2.

The uniqueness from Radon–Nikodým Theorem assures us that $f_{i}\left(\omega ,s\right)=f_{i}^{\left(1\right)}\left(\omega \right)f_{i}^{\left(2\right)}\left(s\right)$ for any ω∈Ω, s∈S and any i=1,2.

Let A∈T and B∈S.

We define the weighted Tsallis divergence for product measures via

$$D_{k}^{w_{1}w_{2},T}\left(\mu_{1}\times \nu_{1}|\mu_{2}\times \nu_{2},A\times B\right)=\;\frac{1}{\underset{A\times B}{\;\int \int \;}w_{1}w_{2}d\left(\mu_{1}\times \nu_{1}\right)}\underset{A\times B}{\;\int \int \;}w_{1}\left(\omega \right)w_{2}\left(s\right)\log_{k}^{T}\left(\frac{f_{1}\left(\omega ,s\right)}{f_{2}\left(\omega ,s\right)}\right)d\left(\mu_{1}\times \nu_{1}\right)\left(\omega ,s\right)=\;\frac{1}{\int_{A}w_{1}d\mu_{1}\int_{B}w_{2}d\nu_{1}}\underset{A\times B}{\;\int \int \;}w_{1}\left(\omega \right)w_{2}\left(s\right)f_{1}\left(\omega ,s\right)\log_{k}^{T}\left(\frac{f_{1}\left(\omega ,s\right)}{f_{2}\left(\omega ,s\right)}\right)d\lambda (\omega ,s)\;\mathrm{if}$$

$$\left(\mu_{1}\times \nu_{1}\right)\left(A\times B\right)\neq 0$$

and

$$D_{k}^{w_{1}w_{2},T}\left(\mu_{1}\times \nu_{1}|\mu_{2}\times \nu_{2},A\times B\right)=0\;\mathrm{if}\;\left(\mu_{1}\times \nu_{1}\right)\left(A\times B\right)=0.$$

The weighted Kaniadakis divergence for product measures is given by

$$D_{k}^{w_{1}w_{2},K}\left(\mu_{1}\times \nu_{1}|\mu_{2}\times \nu_{2},A\times B\right)=\;\frac{1}{\underset{A\times B}{\;\int \int \;}w_{1}w_{2}d\left(\mu_{1}\times \nu_{1}\right)}\underset{A\times B}{\;\int \int \;}w_{1}\left(\omega \right)w_{2}\left(s\right)\log_{k}^{K}\left(\frac{f_{1}\left(\omega ,s\right)}{f_{2}\left(\omega ,s\right)}\right)d\left(\mu_{1}\times \nu_{1}\right)\left(\omega ,s\right)=\;\frac{1}{\int_{A}w_{1}d\mu_{1}\int_{B}w_{2}d\nu_{1}}\underset{A\times B}{\;\int \int \;}w_{1}\left(\omega \right)w_{2}\left(s\right)f_{1}\left(\omega ,s\right)\log_{k}^{K}\left(\frac{f_{1}\left(\omega ,s\right)}{f_{2}\left(\omega ,s\right)}\right)d\lambda (\omega ,s)\;\mathrm{if}$$

$$\left(\mu_{1}\times \nu_{1}\right)\left(A\times B\right)\neq 0$$

and

$$D_{k}^{w_{1}w_{2},K}\left(\mu_{1}\times \nu_{1}|\mu_{2}\times \nu_{2},A\times B\right)=0\;\mathrm{if}\;\left(\mu_{1}\times \nu_{1}\right)\left(A\times B\right)=0.$$

**Lemma** **2** (see [41])**.** *We have the following pseudo-additivity property for Tsallis logarithm (valid for any x,y > 0)*

$$\log_{k}^{T}\left(xy\right)=\log_{k}^{T}x+\log_{k}^{T}y+k\left(\log_{k}^{T}x\right)(\log_{k}^{T}y).$$

**Theorem** **3.** *Let*A∈T and B∈S such that $\left(\mu_{1}\times \nu_{1}\right)\left(A\times B\right)\neq 0$. *The weighted Tsallis and Kaniadakis divergences for product measures satisfy the following pseudo-additivity properties:*

(a) $D_{k}^{w_{1}w_{2},T}\left(\mu_{1}\times \nu_{1}|\mu_{2}\times \nu_{2},A\times B\right)=D_{k}^{w_{1},T}\left(\mu_{1}|\mu_{2},A\right)+D_{k}^{w_{2},T}\left(\nu_{1}|\nu_{2},B\right)+kD_{k}^{w_{1},T}\left(\mu_{1}|\mu_{2},A\right)D_{k}^{w_{2},T}(\nu_{1}|\nu_{2},B).$

(b) $D_{k}^{w_{1}w_{2},K}\left(\mu_{1}\times \nu_{1}|\mu_{2}\times \nu_{2},A\times B\right)=D_{k}^{w_{1},K}\left(\mu_{1}|\mu_{2},A\right)+D_{k}^{w_{2},K}\left(\nu_{1}|\nu_{2},B\right)+\frac{k}{2}\left(D_{k}^{w_{1},T}\left(\mu_{1}|\mu_{2},A\right)D_{k}^{w_{2},T}\left(\nu_{1}|\nu_{2},B\right)-D_{-k}^{w_{1},T}\left(\mu_{1}|\mu_{2},A\right)D_{-k}^{w_{2},T}(\nu_{1}|\nu_{2},B).\right)$

(c) $D_{k}^{w_{1}w_{2},K}\left(\mu_{1}\times \nu_{1}|\mu_{2}\times \nu_{2},A\times B\right)=\frac{1}{\int_{B}w_{2}d\nu_{1}}\left(\int_{B}w_{2}\left(s\right)f_{1}^{\left(2\right)}\left(s\right){\left(\frac{f_{1}^{\left(2\right)}\left(s\right)}{f_{2}^{\left(2\right)}\left(s\right)}\right)}^{k}d\lambda_{2}\left(s\right)\right)\cdot \;D_{k}^{w_{1},K}\left(\mu_{1}|\mu_{2},A\right)+\frac{1}{\int_{A}w_{1}d\mu_{1}}\left(\int_{A}w_{1}\left(\omega \right)f_{1}^{\left(1\right)}\left(\omega \right){\left(\frac{f_{1}^{\left(1\right)}\left(\omega \right)}{f_{2}^{\left(1\right)}\left(\omega \right)}\right)}^{-k}d\lambda_{1}\left(\omega \right)\right)D_{k}^{w_{2},K}(\nu_{1}|\nu_{2},B).$

**Proof** **.** (a) According to Lemma 2, we have

$$D_{k}^{w_{1}w_{2},T}\left(\mu_{1}\times \nu_{1}|\mu_{2}\times \nu_{2},A\times B\right)=\;\frac{1}{\int_{A}w_{1}d\mu_{1}\int_{B}w_{2}d\nu_{1}}\underset{A\times B}{\;\int \int \;}w_{1}\left(\omega \right)w_{2}\left(s\right)f_{1}\left(\omega ,s\right)\log_{k}^{T}\left(\frac{f_{1}\left(\omega ,s\right)}{f_{2}\left(\omega ,s\right)}\right)d\lambda \left(\omega ,s\right)=\;\frac{1}{\int_{A}w_{1}d\mu_{1}}\cdot \frac{1}{\int_{B}w_{2}d\nu_{1}}\underset{A\times B}{\;\int \int \;}w_{1}\left(\omega \right)w_{2}\left(s\right)f_{1}^{\left(1\right)}\left(\omega \right)f_{1}^{\left(2\right)}\left(s\right)\log_{k}^{T}\left(\frac{f_{1}^{\left(1\right)}\left(\omega \right)}{f_{2}^{\left(1\right)}\left(\omega \right)}\right)d\lambda_{1}\left(\omega \right)d\lambda_{2}\left(s\right)+\;\frac{1}{\int_{A}w_{1}d\mu_{1}}\cdot \frac{1}{\int_{B}w_{2}d\nu_{1}}\underset{A\times B}{\;\int \int \;}w_{1}\left(\omega \right)w_{2}\left(s\right)f_{1}^{\left(1\right)}\left(\omega \right)f_{1}^{\left(2\right)}\left(s\right)\log_{k}^{T}\left(\frac{f_{1}^{\left(2\right)}\left(s\right)}{f_{2}^{\left(2\right)}\left(s\right)}\right)d\lambda_{1}\left(\omega \right)d\lambda_{2}\left(s\right)+k\cdot \;\frac{1}{\int_{A}w_{1}d\mu_{1}}\cdot \frac{1}{\int_{B}w_{2}d\nu_{1}}\cdot \;\underset{A\times B}{\;\int \int \;}w_{1}\left(\omega \right)w_{2}\left(s\right)f_{1}^{\left(1\right)}\left(\omega \right)f_{1}^{\left(2\right)}\left(s\right)\left(\log_{k}^{T}\left(\frac{f_{1}^{\left(1\right)}\left(\omega \right)}{f_{2}^{\left(1\right)}\left(\omega \right)}\right)\right)\left(\log_{k}^{T}\left(\frac{f_{1}^{\left(2\right)}\left(s\right)}{f_{2}^{\left(2\right)}\left(s\right)}\right)\right)d\lambda_{1}\left(\omega \right)d\lambda_{2}\left(s\right)=\;\frac{1}{\int_{A}w_{1}d\mu_{1}}\left(\int_{A}w_{1}\left(\omega \right)f_{1}^{\left(1\right)}\left(\omega \right)\log_{k}^{T}\left(\frac{f_{1}^{\left(1\right)}\left(\omega \right)}{f_{2}^{\left(1\right)}\left(\omega \right)}\right)d\lambda_{1}\left(\omega \right)\right)\frac{1}{\int_{B}w_{2}d\nu_{1}}\cdot \;\left(\int_{B}w_{2}\left(s\right)f_{1}^{\left(2\right)}\left(s\right)d\lambda_{2}\left(s\right)\right)+\;\frac{1}{\int_{A}w_{1}d\mu_{1}}\left(\int_{A}w_{1}\left(\omega \right)f_{1}^{\left(1\right)}\left(\omega \right)d\lambda_{1}\left(\omega \right)\right)\frac{1}{\int_{B}w_{2}d\nu_{1}}\left(\int_{B}w_{2}\left(s\right)f_{1}^{\left(2\right)}\left(s\right)\log_{k}^{T}\left(\frac{f_{1}^{\left(2\right)}\left(s\right)}{f_{2}^{\left(2\right)}\left(s\right)}\right)d\lambda_{2}\left(s\right)\right)+\;k\cdot \frac{1}{\int_{A}w_{1}d\mu_{1}}\left(\int_{A}w_{1}\left(\omega \right)f_{1}^{\left(1\right)}\left(\omega \right)\log_{k}^{T}\left(\frac{f_{1}^{\left(1\right)}\left(\omega \right)}{f_{2}^{\left(1\right)}\left(\omega \right)}\right)d\lambda_{1}\left(\omega \right)\right)\frac{1}{\int_{B}w_{2}d\nu_{1}}\cdot \;\left(\int_{B}w_{2}\left(s\right)f_{1}^{\left(2\right)}\left(s\right)\log_{k}^{T}\left(\frac{f_{1}^{\left(2\right)}\left(s\right)}{f_{2}^{\left(2\right)}\left(s\right)}\right)d\lambda_{2}\left(s\right)\right)=\;D_{k}^{w_{1},T}\left(\mu_{1}|\mu_{2},A\right)+D_{k}^{w_{2},T}\left(\nu_{1}|\nu_{2},B\right)+kD_{k}^{w_{1},T}\left(\mu_{1}|\mu_{2},A\right)D_{k}^{w_{2},T}(\nu_{1}|\nu_{2},B).$$

(b) Because $\log_{k}^{K}x=\frac{1}{2}\left(\log_{k}^{T}x+\log_{-k}^{T}x\right)$, we have

$$D_{k}^{w_{1}w_{2},K}\left(\mu_{1}\times \nu_{1}|\mu_{2}\times \nu_{2},A\times B\right)=\;\frac{1}{\int_{A}w_{1}d\mu_{1}\int_{B}w_{2}d\nu_{1}}\underset{A\times B}{\;\int \int \;}w_{1}\left(\omega \right)w_{2}\left(s\right)f_{1}\left(\omega ,s\right)\log_{k}^{K}\left(\frac{f_{1}\left(\omega ,s\right)}{f_{2}\left(\omega ,s\right)}\right)d\lambda \left(\omega ,s\right)=\;\frac{1}{\int_{A}w_{1}d\mu_{1}}\cdot \frac{1}{\int_{B}w_{2}d\nu_{1}}\cdot \underset{A\times B}{\;\int \int \;}w_{1}\left(\omega \right)w_{2}\left(s\right)f_{1}^{\left(1\right)}\left(\omega \right)f_{1}^{\left(2\right)}\left(s\right)\cdot \;\frac{1}{2}\left(\log_{k}^{T}\left(\frac{f_{1}^{\left(1\right)}\left(\omega \right)f_{1}^{\left(2\right)}\left(s\right)}{f_{2}^{\left(1\right)}\left(\omega \right)f_{2}^{\left(2\right)}\left(s\right)}\right)+\log_{-k}^{T}\left(\frac{f_{1}^{\left(1\right)}\left(\omega \right)f_{1}^{\left(2\right)}\left(s\right)}{f_{2}^{\left(1\right)}\left(\omega \right)f_{2}^{\left(2\right)}\left(s\right)}\right)\right)d\lambda_{1}\left(\omega \right)d\lambda_{2}\left(s\right)=\;\frac{1}{2}\left(D_{k}^{w_{1}w_{2},T}\left(\mu_{1}\times \nu_{1}|\mu_{2}\times \nu_{2},A\times B\right)+D_{-k}^{w_{1}w_{2},T}(\mu_{1}\times \nu_{1}|\mu_{2}\times \nu_{2},A\times B).\right)$$

Using (a), we get

$$D_{k}^{w_{1}w_{2},K}\left(\mu_{1}\times \nu_{1}|\mu_{2}\times \nu_{2},A\times B\right)=\;\frac{1}{2}\left(D_{k}^{w_{1},T}\left(\mu_{1}|\mu_{2},A\right)+D_{k}^{w_{2},T}\left(\nu_{1}|\nu_{2},B\right)+kD_{k}^{w_{1},T}\left(\mu_{1}|\mu_{2},A\right)D_{k}^{w_{2},T}\left(\nu_{1}|\nu_{2},B\right)\right)+\;\frac{1}{2}\left(D_{-k}^{w_{1},T}\left(\mu_{1}|\mu_{2},A\right)+D_{-k}^{w_{2},T}\left(\nu_{1}|\nu_{2},B\right)-kD_{-k}^{w_{1},T}\left(\mu_{1}|\mu_{2},A\right)D_{-k}^{w_{2},T}\left(\nu_{1}|\nu_{2},B\right)\right)=\;D_{k}^{w_{1},K}\left(\mu_{1}|\mu_{2},A\right)+D_{k}^{w_{2},K}\left(\nu_{1}|\nu_{2},B\right)+\;\frac{k}{2}\left(D_{k}^{w_{1},T}\left(\mu_{1}|\mu_{2},A\right)D_{k}^{w_{2},T}\left(\nu_{1}|\nu_{2},B\right)-D_{-k}^{w_{1},T}\left(\mu_{1}|\mu_{2},A\right)D_{-k}^{w_{2},T}\left(\nu_{1}|\nu_{2},B\right)\right).$$

(c) It is easy to prove that

$$\log_{k}^{K}\left(xy\right)=y^{k}\cdot \frac{x^{k}-x^{-k}}{2k}+x^{-k}\cdot \frac{y^{k}-y^{-k}}{2k}=y^{k}\log_{k}^{K}x+x^{-k}\log_{k}^{K}y\;\mathrm{for}\;\mathrm{any}\;x,y\in (0,\infty ).$$

Hence,

$$D_{k}^{w_{1}w_{2},K}\left(\mu_{1}\times \nu_{1}|\mu_{2}\times \nu_{2},A\times B\right)=\;\frac{1}{\int_{A}w_{1}d\mu_{1}\int_{B}w_{2}d\nu_{1}}\underset{A\times B}{\;\int \int \;}w_{1}\left(\omega \right)w_{2}\left(s\right)f_{1}\left(\omega ,s\right)\log_{k}^{K}\left(\frac{f_{1}\left(\omega ,s\right)}{f_{2}\left(\omega ,s\right)}\right)d\lambda \left(\omega ,s\right)=\;\frac{1}{\int_{A}w_{1}d\mu_{1}}\cdot \frac{1}{\int_{B}w_{2}d\nu_{1}}\underset{A\times B}{\;\int \int \;}w_{1}\left(\omega \right)w_{2}\left(s\right)f_{1}^{\left(1\right)}\left(\omega \right)f_{1}^{\left(2\right)}\left(s\right)\left[{\left(\frac{f_{1}^{\left(2\right)}\left(s\right)}{f_{2}^{\left(2\right)}\left(s\right)}\right)}^{k}\log_{k}^{K}\left(\frac{f_{1}^{\left(1\right)}\left(\omega \right)}{f_{2}^{\left(1\right)}\left(\omega \right)}\right)+\;{\left(\frac{f_{1}^{\left(1\right)}\left(\omega \right)}{f_{2}^{\left(1\right)}\left(\omega \right)}\right)}^{-k}\log_{k}^{K}\left(\frac{f_{1}^{\left(2\right)}\left(s\right)}{f_{2}^{\left(2\right)}\left(s\right)}\right)\right]d\lambda_{1}\left(\omega \right)d\lambda_{2}\left(s\right)=\;\frac{1}{\int_{A}w_{1}d\mu_{1}}\left(\int_{A}w_{1}\left(\omega \right)f_{1}^{\left(1\right)}\left(\omega \right)\log_{k}^{K}\left(\frac{f_{1}^{\left(1\right)}\left(\omega \right)}{f_{2}^{\left(1\right)}\left(\omega \right)}\right)d\lambda_{1}\left(\omega \right)\right)\cdot \;\frac{1}{\int_{B}w_{2}d\nu_{1}}\left(\int_{B}w_{2}\left(s\right)f_{1}^{\left(2\right)}\left(s\right){\left(\frac{f_{1}^{\left(2\right)}\left(s\right)}{f_{2}^{\left(2\right)}\left(s\right)}\right)}^{k}d\lambda_{2}\left(s\right)\right)+\;\frac{1}{\int_{A}w_{1}d\mu_{1}}\left(\int_{A}w_{1}\left(\omega \right)f_{1}^{\left(1\right)}\left(\omega \right){\left(\frac{f_{1}^{\left(1\right)}\left(\omega \right)}{f_{2}^{\left(1\right)}\left(\omega \right)}\right)}^{-k}d\lambda_{1}\left(\omega \right)\right)\cdot \;\frac{1}{\int_{B}w_{2}d\nu_{1}}\left(\int_{B}w_{2}\left(s\right)f_{1}^{\left(2\right)}\left(s\right)\log_{k}^{K}\left(\frac{f_{1}^{\left(2\right)}\left(s\right)}{f_{2}^{\left(2\right)}\left(s\right)}\right)d\lambda_{2}\left(s\right)\right)=\;\frac{1}{\int_{B}w_{2}d\nu_{1}}\left(\int_{B}w_{2}\left(s\right)f_{1}^{\left(2\right)}\left(s\right){\left(\frac{f_{1}^{\left(2\right)}\left(s\right)}{f_{2}^{\left(2\right)}\left(s\right)}\right)}^{k}d\lambda_{2}\left(s\right)\right)D_{k}^{w_{1},K}\left(\mu_{1}|\mu_{2},A\right)+\;\frac{1}{\int_{A}w_{1}d\mu_{1}}\left(\int_{A}w_{1}\left(\omega \right)f_{1}^{\left(1\right)}\left(\omega \right){\left(\frac{f_{1}^{\left(1\right)}\left(\omega \right)}{f_{2}^{\left(1\right)}\left(\omega \right)}\right)}^{-k}d\lambda_{1}\left(\omega \right)\right)D_{k}^{w_{2},K}(\nu_{1}|\nu_{2},B).$$

□

## 5. Conclusions

With the help of some inequalities concerning Tsallis logarithm, we obtained inequalities between the weighted Tsallis and Kaniadakis divergences on an arbitrary measurable non-negligible set and Tsallis logarithm, respectively, Kaniadakis logarithm (Theorem 1). We showed that the aforementioned divergences are limited by similar bounds with those that limit Kullback–Leibler divergence (Theorem 2) and proved that are pseudo-additive (Theorem 3). 

## Data Availability

Not applicable.
